# The variations on the aerodynamics of a world-ranked wheelchair sprinter in the key-moments of the stroke cycle: A numerical simulation analysis

**DOI:** 10.1371/journal.pone.0193658

**Published:** 2018-02-28

**Authors:** Pedro Forte, Daniel A. Marinho, Jorge E. Morais, Pedro G. Morouço, Tiago M. Barbosa

**Affiliations:** 1 Department of Sport Sciences, University of Beira Interior, Covilhã, Portugal; 2 Research Centre in Sports, Health and Human Development, Covilhã, Portugal; 3 Department of Sport Sciences, Polytechnic Institute of Bragança, Bragança, Portugal; 4 Department of Sport Sciences, Polytechnic Institute of Leiria, Leiria, Portugal; 5 Centre for Rapid and Sustainable Product Development, Leiria, Portugal; 6 National Institute of Education, Nanyang Technological University, Singapore, Singapore; University of Illinois at Urbana-Champaign, UNITED STATES

## Abstract

Biomechanics plays an important role helping Paralympic sprinters to excel, having the aerodynamic drag a significant impact on the athlete’s performance. The aim of this study was to assess the aerodynamics in different key-moments of the stroke cycle by Computational Fluid Dynamics. A world-ranked wheelchair sprinter was scanned on the racing wheelchair wearing his competition gear and helmet. The sprinter was scanned in three different positions: (i) catch (hands in the 12h position on the hand-rim); (ii) the release (hands in the 18h position on the hand-rim) and; (iii) recovery phase (hands do not touch the hand-rim and are hyperextended backwards). The simulations were performed at 2.0, 3.5, 5.0 and 6.5 m/s. The mean viscous and pressure drag components, total drag force and effective area were retrieved after running the numerical simulations. The viscous drag ranged from 3.35 N to 2.94 N, pressure drag from 0.38 N to 5.51 N, total drag force from 0.72 N to 8.45 N and effective area from 0.24 to 0.41 m^2^. The results pointed out that the sprinter was submitted to less drag in the recovery phase, and higher drag in the catch. These findings suggest the importance of keeping an adequate body alignment to avoid an increase in the drag force.

## Introduction

Wheelchair sprinting events are some of the most popular races in Paralympics. In these events, athletes compete on racing wheelchairs designed to let them reach their maximal speed [1].

Biomechanics plays an important role helping Paralympic sprinters to excel. The proper alignment of the body segments and a good stroke technique will help to reduce the winning time [2]. To reach the maximal acceleration as soon as possible and maintain a maximal speed over the race, the resistive forces must be minimized and propulsive forces maximized [2, 3]. The propulsive forces (pushing the hand-rim and the wheels producing force on the ground), should overcome the resistive forces (i.e., the rolling friction and aerodynamic drag). Each stroke cycle can be broken-down into three key-moments. The first moment is the beginning of the propulsive phase, being the hands in the 12h position on the hand-rim (known as catch phase). When the hands are in the 18h position on the hand-rim, the propulsion phase ends (known as release phase). When the hands are not in contact with the hand-rim and hyperextended backwards, this is known as the recovery phase [2]. The propulsive phases (between catch and release) account for approximately 35% of the stroke cycle´s duration and the recovery the remaining 65% [2].

In wheelchair racing, aerodynamic drag has a significant impact on the performance at speeds higher than 5 m/s [4–8]. At world record pace, drag force may account for 34.89% of the overall resistive forces [9]. However, no study was carried out comparing the aerodynamics of a sprinter in the key-moments of the stroke cycle (i.e. catch, release and recovery phases).

It was claimed that marginal shifts in the rider’s position on the wheelchair might change the drag by 10% [10–12]. Still, there is scarce evidence on this. Barbosa et al. [13] compared by experimental testing the resistive forces in three different head and torso positions, noting variations in the aerodynamics. Thus, the drag force may change in the key-moments of the stroke cycle depending on the relative position of the upper arms, torso and head. E.g., the change in the relative position may have influence on the surface area and, hence, on the drag coefficient.

Numerical simulations by Computational Fluid Dynamics (CFD) are arguably the best technique available to run this comparison. Benchmarked with other procedures to monitor the aerodynamics, CFD has the advantage of minimizing confounding factors such as the intra-subject variability and the changes in the environmental conditions across trials. It is possible to control with higher accuracy the temperature, pressure and speed of each simulation. Unfortunately, the control of these factors in experimental tests is more challenging. Besides that, using CFD it is possible to breakdown the total drag force into viscous and pressure drag [2].

Pressure drag can be characterised as the fluid distortion in the rear edges and the pressure differences between the front and back boundaries of the body (in our study the wheelchair-athlete system) [14, 15]. The fluid separation from the back boundaries will generate a low pressure zone, mainly caused by the object/body shape [14, 15]. The pressure drag is dependent on several factors, including the body size and geometry [15–20]. As such, arguably in wheelchair racing, the athlete’s position will influence the pressure drag. Nevertheless, it was not found any evidence on this in the literature.

Viscous drag is produced due to the interaction between the body’s surface and the fluid [14]. In the case of a fluid with viscosity, such as air, the fluid is going to stick to the body’s surface and being dragged. Because of the viscosity, this layer of fluid attached to the body will make the following layer to attach to itself. Same phenomenon happens to nearby layers. Viscous drag is the force needed to drag the sum of all layers of fluid. Thus, this component is strongly dependent on the speed and surfaces roughness. Although viscous drag might have arguably a smaller impact compared to pressure drag, it has important implications on the athlete’s performance. The body position, garments’ design and materials used, as well as, surface’s roughness will have an effect on viscous drag [14, 21]. There are claims that in wheelchair racing, viscous drag can be decreased by reducing the surface roughness, for instance, wearing light and tight garments. However, no study was founded assessing viscous drag in wheelchair racing.

Effective aerodynamic area (ACd) is a well-rounded parameter to assess the aerodynamics of a body. It is obtained by the multiplication of the drag coefficient by the surface area. ACd is the area that acts in the drag-production direction (opposite direction of the flow) [14, 22]. E.g., in cycling, time trial positions are recommended to decrease the ACd [22, 23]. Thus, ACd is mainly dependent of the athlete’s surface area and position. Some studies monitored the ACd of wheelchair sprinters [13, 24]. Barbosa et al., [13] observed values between 0.1456m^2^ and 0.1747m^2^; whereas, Hoffman at el., [24] reported an ACd of 0.37m^2^. The data reported by these authors are mean values for the entire stroke cycle. There is no evidence on the changes in the ACd in different key-moments of the stroke cycle in wheelchair sprinting.

The aim of this study was to assess the aerodynamics in different key-moments of the stroke cycle by CFD. It was hypothesised that the drag varies in different phases of the stroke cycle, depending on the relative position of the segments.

## Methods

### Subject

A world-ranked wheelchair sprinter (ranked 4^th^ in the 100m and 400m T-52 category at the time of the data collection) volunteered to take part of this study.

Approval was granted by the Ethics Committee of the University of Beira Interior. All procedures were in accordance to the Helsinki Declaration regarding human research. A written consent by the participant was obtained beforehand.

### Scanning the model

**Fig 1:**
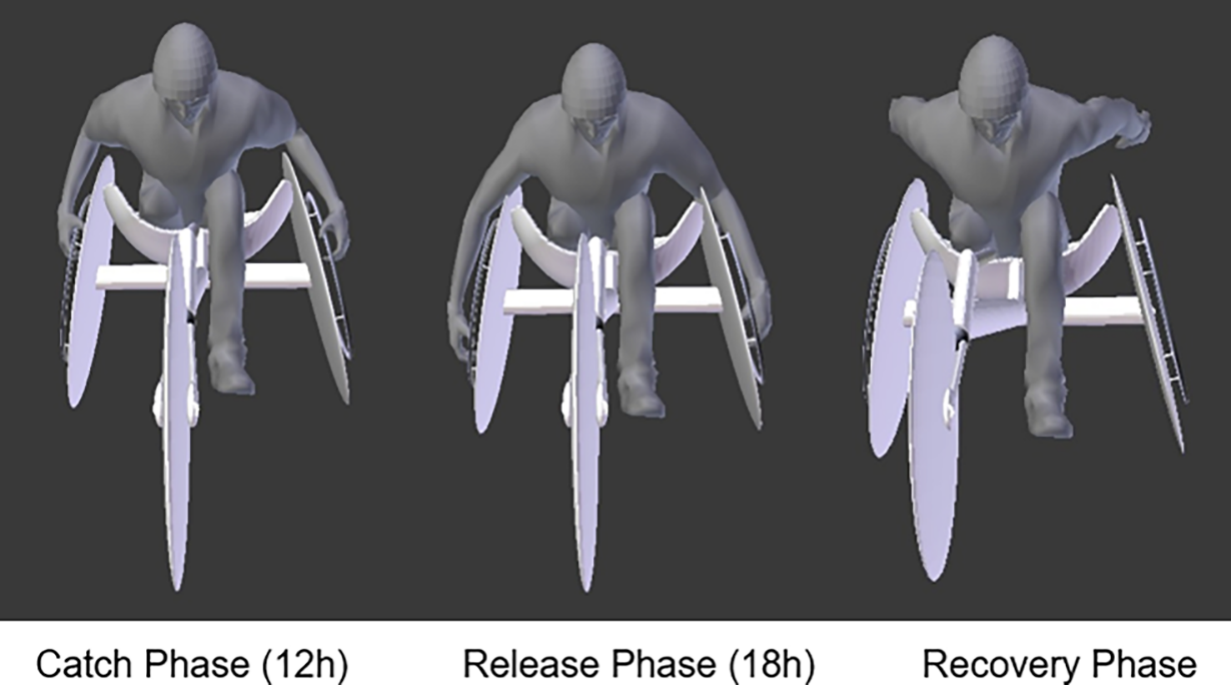
Three different scanned positions: (i) catch (i.e., the beginning of the propulsive phase, being the hands in the 12h position on the hand-rim); (ii) the release (i.e., hands in the 18h position on the hand-rim) and; (iii) recovery phase (i.e., hands do not touch the hand-rim and are hyperextended backwards) respectively.

The sprinter was scanned on the racing wheelchair wearing his competition gear and helmet. The scans were made in three different positions (Fig 1): (i) catch (i.e., the beginning of the propulsive phase, being the hands in the 12h position on the hand-rim); (ii) the release (i.e., hands in the 18h position on the hand-rim) and; (iii) recovery phase (i.e., hands do not touch the hand-rim and are hyperextended backwards). The 12h position is set when the hand catches the hand-rim (the catch phase). In this position, the hands contact the hand-rim in an angle of about 0° to 15° with the vertical axis (i.e. on the top of the wheel). The 18h position is set when the hands leave the hand-rim (release phase). The hand contact breaks off, usually near the 180° angle with the vertical axis (i.e. on the bottom of the wheel).

The scanning was made by the 3D Artec Scanner (Artec Group, Inc., Luxembourg) and saved in the Artec Studio 0.7 (Artec Group, Inc., USA). The same software was used to edit the scans (e.g., smooth and merge all the scan layers). Upon that, Geomagic studio (3D Systems, USA) was used to mesh the object and improve it by smoothing self-intersections, clean noun-manifold edges, and spikes correction being then converted in a computer aided design (CAD) model.

### Numerical simulation

The CAD models were imported into Fluent CFD code (Ansys Fluent 16.0, Ansys Inc., Pennsylvania, USA). The numerical simulation was underpinned by the discretization of the Navier-Stokes equations by the finite volumes methods. The Reynolds-Averaged Navier-Stokes converts instantaneous values into means. Fluent CFD code (Ansys Fluent 16.0, Ansys Inc., Pennsylvania, USA.) allowed to solve these equations using the finite volume approach, having the equations been integrated over each control volume. The behaviour of the fluid flow (Eq 1), Reynolds stresses (Eq 2), temperature (Eq 3) and mass transfer (Eq 4) have been solved as follows:

$$\frac{\partial_{Ui}}{\partial_{xi}}=0$$ (1)

$$\frac{\partial_{Ui}}{\partial_{t}}\pm U_{j}\frac{\partial_{Ui}}{\partial_{x_{j}}}=-\frac{1}{\rho }\frac{\partial P}{{\partial x}_{j}}+\frac{\partial }{{\partial x}_{j}}(2vS_{ij}-\bar{\mu_{j}\prime \mu_{i}\prime })$$ (2)

$$\frac{\partial_{\theta i}}{\partial_{t}}\pm U_{j}\frac{\partial_{\theta }}{\partial_{x_{j}}}=\frac{1}{\rho_{cp}}\frac{\partial }{{\partial x}_{j}}\left(k\frac{\partial \theta }{{\partial x}_{j}}-\bar{\mu_{j}\prime \theta \prime }\right)$$ (3)

$$\frac{\partial_{C}}{\partial_{t}}\pm U_{j}\frac{\partial_{C}}{\partial_{x_{j}}}=\frac{\partial }{{\partial x}_{j}}\left(D\frac{\partial C}{{\partial x}_{j}}-\bar{\mu_{j}\prime c\prime }\right)$$ (4)

Where, μ_i_ and x_i_ are the instantaneous velocity and the position, p is the instantaneous pressure, t is the time, ρ is the fluid density, v is the molecular kinematic viscous, cp is the heat capacity, k is the thermal conductivity, S_ij_ is the strain-rate tensor, c is the instantaneous concentration, and D is the molecular diffusion coefficient. The Reynolds stresses component ($\bar{\mu_{j}\prime \mu_{i}\prime }$), describes the turbulence of the mean flow being the exchange of momentum by the change of the fluid parcels.

The *realizable k-epsilon* was the turbulence model selected for this research. This model presents velocity histograms similar to *standard k-e*, *RST and RNG k-e* models. The latter models converged after 11876, 3208 and 2874 interactions, respectively. However, the *realizable k-epsilon* only required 1404 interactions to converge the solution, therefore, showing a higher computation economy [25]. The aerodynamic drag force was computed as:

$$F_{D}=0.5\rho A_{d}v^{2}C_{D}$$ (5)

Where F_D_ is the drag force, C_D_ represents the drag coefficient, v the relative velocity, A_d_ surface area and ρ is the fluid density.

### Boundary conditions

**Fig 2:**
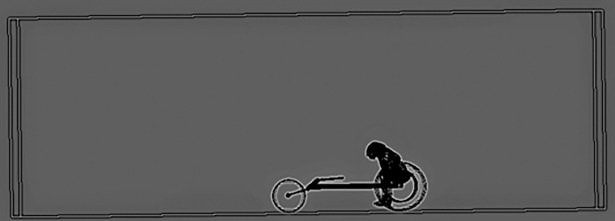
Wheelchair-athlete system in the enclosure.

The three-dimensional domain was meshed to depict the fluid flow around the athlete-wheelchair system (Fig 2). The whole domain (3m x 2m x 1.5m) was composed by 35 million of prisms and pyramids elements and created in the Ansys meshing module (Ansys Inc., Pennsylvania, USA).

To create an accurate model, the grid node separation in areas of high velocity and pressure was decreased [26, 27]. The subtract operation was used to separate the wheelchair-athlete from the enclosure and define it as an object inside the tunnel. This procedure was carried out for the three different positions (catch, release and recovery phases). The body of the athlete-wheelchair system was aligned with the z-axis direction.

The air velocity was set in the inlet portion of the dome surface (-z direction), with steady values between 2.0 and 6.5 m/s (increments of 1.5 m/s in each simulation). The turbulence intensity was set at 1×10^−6^%. The surface of the sprinter was modelled as a non-slip wall with zero roughness, at which scalable wall functions were assigned. The SIMPLE algorithm scheme was selected to solve the pressure-velocity coupling. For the spatial discretization, the Green-Gauss cell-based gradient was chosen [28]. Pressure and momentum were defined as second order and second order upwind, respectively. Turbulent kinetic energy and turbulent dissipation rate were set as first order upwind.

### Outcomes

The mean viscous and pressure drag components, total drag coefficient and total drag force were retrieved. The surface area was obtained by Geomagic studio (3D Systems, USA) and then the ACd computed.

## Results

**Fig 3:**
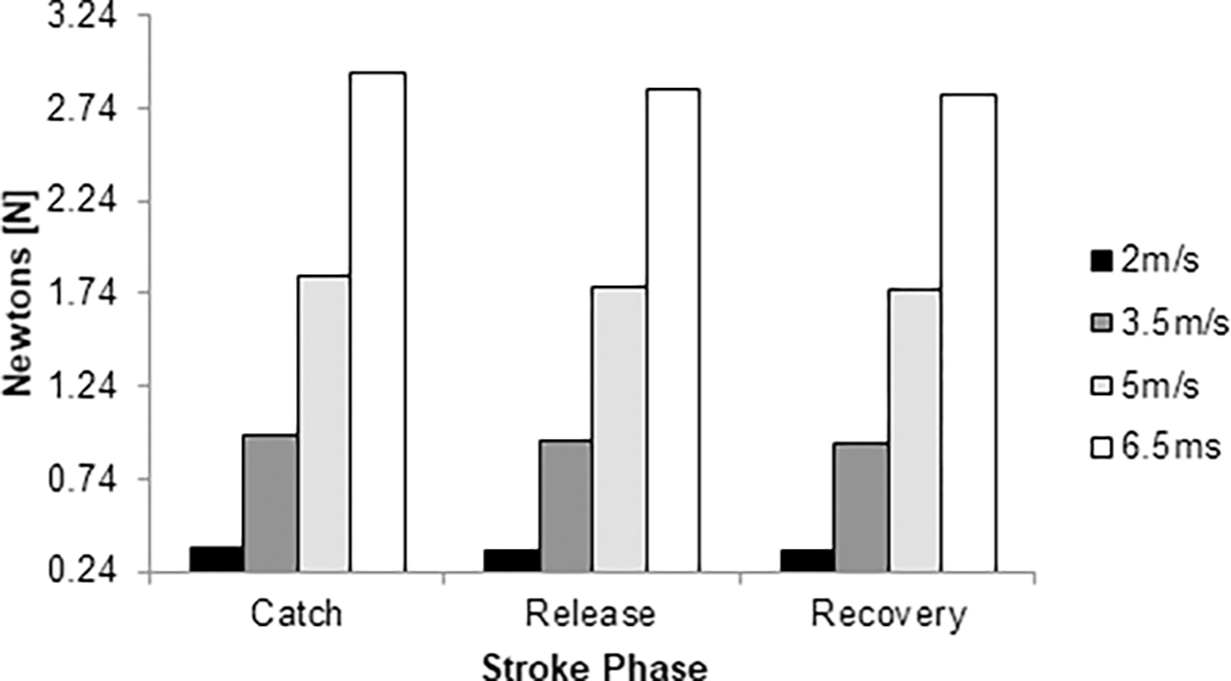
Viscous drag over the stroke cycle at 2.0 m/s (black column), 3.5m/s (dark grey column), 5.0 m/s (light grey column) and 6.5 m/s (white column).

Viscous drag force ranged between 0.35 N and 2.94 N, from 2.0 m/s to 6.5 m/s, respectively (Fig 3). The highest magnitude was noted in the catch phase and the lowest in the recovery phase. The difference between catch and release phases was 3–4% across the selected speeds. The release and recovery phases differed by 1–2%. The differences between the catch and recovery phases were 3–4%. Thus, the differences between key-moments of the stroke cycle ranged between 1% and 4%.

**Fig 4:**
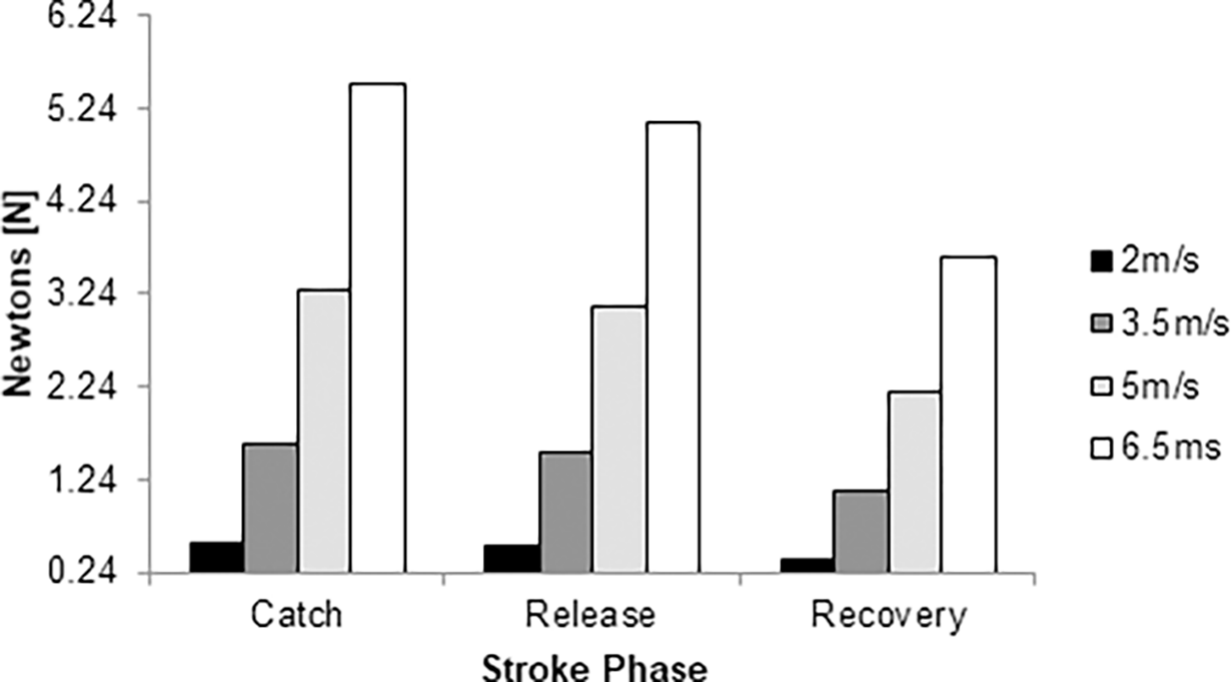
Pressure drag force over the stroke cycle at 2.0 m/s (black column), 3.5m/s (dark grey column), 5.0 m/s (light grey column) and 6.5 m/s (white column).

The pressure drag ranged between 0.38 N and 5.51 N for the same speed range (Fig 4). The highest magnitude was noted in the catch phase; whereas, the lowest values in the recovery phase. The differences between catch and release phases were about 3% to 8% across the selected speeds. From the release to recovery phases, the differences ranged from 37% to 43%. The catch phase differed in 43% to 52% from the catch phase. Therefore, the pressure drag differed about 3% to 52% over the entire stroke cycle.

**Fig 5:**
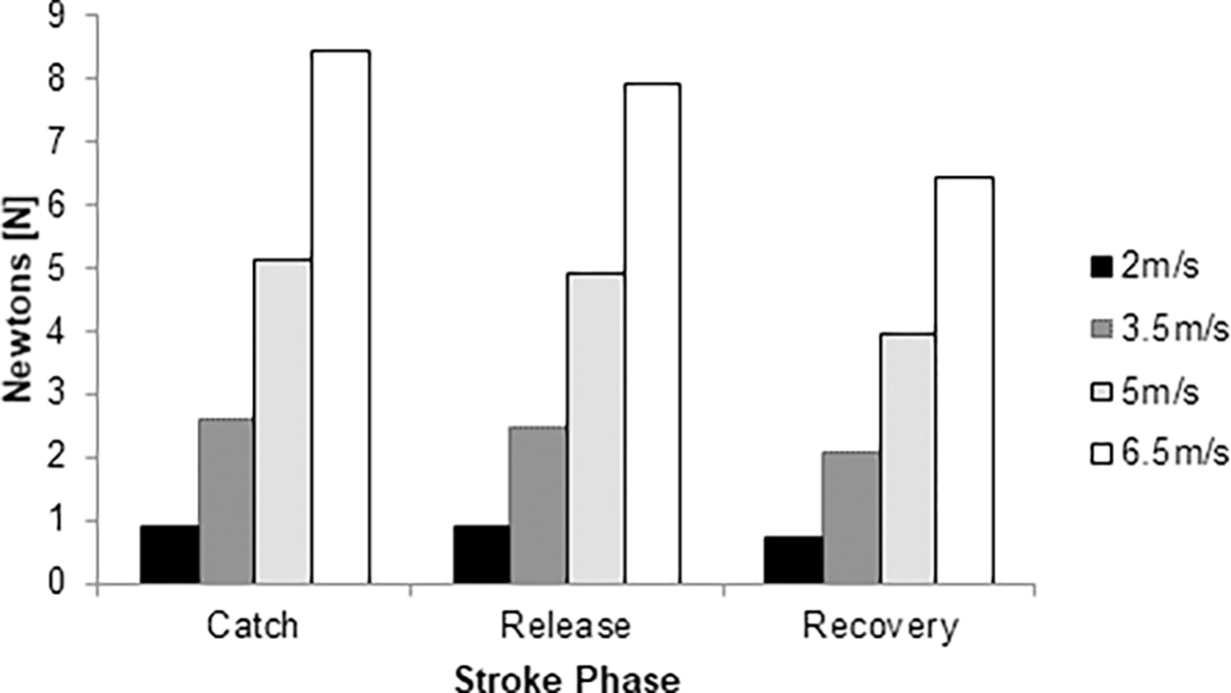
Total drag force over the stroke cycle at 2.0 m/s (black column), 3.5m/s (dark grey column), 5.0 m/s (light grey column) and 6.5 m/s (white column).

The total drag force ranged between 0.72 N and 8.45 N (Fig 5). The key-moment under less drag was the recovery, and the catch phase the highest. At the selected speeds, it was observed a decreasing drag from the catch to the release phase (3–7%). Between the release and recovery phase, total drag decreased between 21% and 24%. The catch phase differs from the recovery by 25–31%.

**Fig 6:**
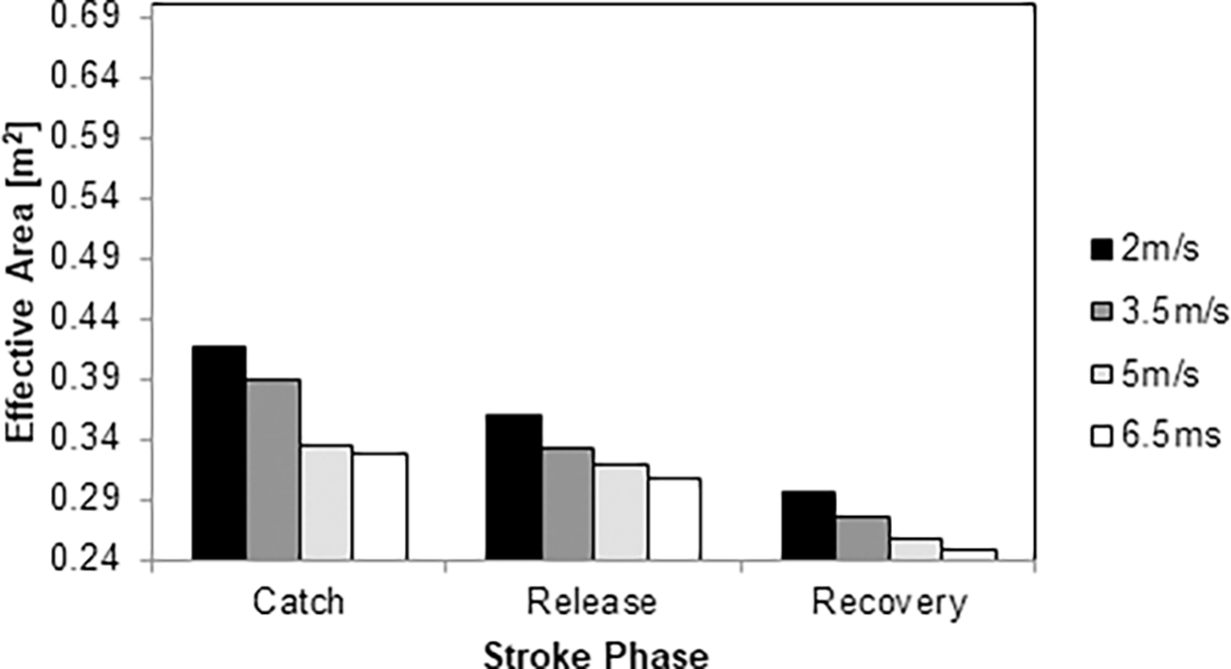
Effective area over the stroke cycle at 2.0 m/s (black column), 3.5m/s (dark grey column), 5.0 m/s (light grey column) and 6.5 m/s (white column).

The ACd ranged from 0.24 to 0.41 m^2^ across the selected speeds. The lowest value was noted in the recovery phase and the highest in the catch phase (Fig 6). From the catch to the release phases, the ACd decreased between 7% and 17%. From the release to the recovery phase, the difference was about 21–24%. The differences between the recovery and catch phases were 30–41%. Altogether, the best ACd was of the three key-moments was found in the recovery phase.

## Discussion

The aim of this study was to compare the aerodynamics in three key-moments of the stroke cycle in wheelchair racing by CFD. The recovery phase was the most aerodynamic position, followed-up by the release and catch phases.

As above-mentioned, no research has been conducted assessing the aerodynamics of wheelchair sprinters by CFD. However, Forte et al. [1] performed a numerical simulation of a road helmet in two different positions at speeds slower than 6.5m/s (near the typical speed reached by wheelchair sprinters in the T52 category). The ACd ranged from 0.024 to 0.034 m^2^. The most aerodynamic position of the head and helmet was keeping an angle of attack of 0° (looking forward). Several studies can be found in the literature monitoring high-speed vehicles in other sports. Winkler and Pernpeintner [29] tested the brakeman’s (behind the pilot) arms position in bobsleigh. The authors reported that the bent arms position showed an ACd of 0.0596m^2^ and with the arms stretched holding the sidewalls of the bobsleigh 0.0609m^2^. The authors noted that the arms’ positions influenced by about 2% the ACd. Defraeye et al. [30] performed a numerical simulation of a cyclist in three different positions. The ACd ranged from 0.169m^2^ to 0.235m^2^. Other studies were performed evaluating the cyclists’ ACd [31–34]. Blocken et al., [31] performed an analysis of two cyclists in three different positions (upright, dropped and time-trial position) between 60 and 100km/h. The authors reported an ACd between 0.131m^2^ and 0.211m^2^. Time-trial was the most aerodynamic position. In this position the cyclists were keeping a flexed trunk and looking forward. Griffith et al. [32] assessed the effect of the leg position on the ACd. The ACd ranged from 0.16m^2^ to 0.24m^2^ keeping symmetric and asymmetric knees alignment. The asymmetric knees alignment (one leg fully extended and the other flexed and raised close to the torso) presented the highest ACd.

The values for viscous drag ranged from 3.35 N to 2.94 N. There are no reports about viscous drag in wheelchair racing that we can use to benchmark our data. It is possible to reduce viscous drag by decreasing the surface roughness, for instance, wearing specific garment. Viscous drag in cycling is about 5% of total drag [30]. In our study, the viscous drag ranged from 35% to 49% of total drag. The higher contribution by viscous component to total drag in wheelchair racing in comparison to cycling can be due to: (i) maximal speed in the T52 category is about 6.5 m/s (i.e., 23.40 km/h). In cycling, performers can reach a higher speed. Studies are conducted at speeds over 60km/h [31] and; (ii) the surface area of an athlete-wheelchair system is larger than a bicycle-athlete system. Viscous drag is strongly dependent on the surface area, where a higher area will lead to a higher magnitude of this force. In the 12h arms position, viscous drag ranged from 0.36 N to 2.93 N. In the 18h arms position, the viscous drag varied from 0.35 N to 2.83 N. In the recovery phase, viscous drag values were between 0.34N and 2.81 N. In the 12h arms position it was noted the largest ACd. On top of that, the arms flection may had increased the surface roughness. In the 18h position, the arms were fully stretched downwards and the surface roughness might have decreased. This roughness can be due to wrinkles and folded tissue in the racing suit. Over the recovery phase, the arms are overstretched backwards and the trunk flexed forward. In this position, arguably the surface might have had a lower roughness because the arms are hyperextended backwards.

Pressure drag ranged between 0.38 N and 5.51 N. The total drag ranged between 0.72 N and 8.45 N. In cycling, the pressure drag accounts for 90% of the total drag. Apparel, such as helmets, may help to reduce pressure drag [1]. Authors also reported that pressure drag is the main contributor for total drag in cycling, running and swimming [8, 35, 36]. In our study, pressure drag had a contribution of 51–65% to total drag. Again, this might be caused by the wheelchair-athlete surface area and the selected speeds. At higher speeds, pressure drag is prone to increase meaningfully. In this study, pressure drag varied in 12h arms position between 0.54 N and 5.51 N. In the 18h position, the differences were between 0.52 N and 5.09 N. In the recovery phase pressure drag values ranged between 0.34 N and 2.91 N. Pressure drag is generated by the pressure differences between the front and back boundaries of the body (in our case the wheelchair-athlete system) [15–20]. The recovery phase had the lowest pressure drag because the sprinter kept the upper-arms hyperextended backwards. In this position, the total length of the wheelchair-athlete system (length from the front wheel to the tip of the fingers extended backwards in the horizontal plane) increases slightly. Moreover, the geometry of the system is modified, keeping a more aerodynamic position (lower angle of attack by the upper-body) and decreasing the ACd. On the other side, in the 18h position the overall geometry of the system increases the surface area. The upper-body is in an upright position and the upper-arms fully extended and facing downwards, increasing the area exposed on the direction of displacement.

The ACd values ranged from 0.24 to 0.41 m^2^ across the selected speeds. Barbosa et al. [13] noted an ACd of about 0.15 m^2^ in one elite wheelchair racer. The authors tested aerodynamics by coast-down technique. Hoffman et al., [24] tested five different wheelchairs also by the coast-down technique, noting a value of 0.37 m^2^. Our findings (ACd: 0.24–0.41m^2^) are in alignment with these latter results. In our study, ACd varied in the 12h position from 0.32 m^2^ to 0.41 m^2^. In the 18h position, ranged between 0.31 m^2^ and 0.35 m^2^. In the recovery phase the ACd values were between 0.24 m^2^ and 0.29 m^2^. The ACd is the wheelchair-athlete system area that acts in the drag-production direction [23]. Thus, it is possible to argue that the different positions influenced the drag force [22, 23]. The 12h arms position presented the highest drag-production area. In the 18h arms position, the trunk flection and the arms near the hand-rims seem to explain the decrease in the drag-production. The same might had occur in the recovery phase; despite the trunk flection and the arms in a hyperextended backwards position decreased ACd and the drag-production area. The drag coefficient (and as such the ACd of our sprinter) is affected by Reynolds number. In this study, our subject showed a Reynolds number of 2.82×10^5^, 4.93×10^5^, 7.04×10^5^ and 9.15×10^5^ at 2.0, 3.5, 5.0 and 6.5 m/s, respectively. For several bodies it is noted a significant drop in the drag coefficient at Reynolds number of 10^5^ [14, 37]. E.g., in a sphere the drag coefficient decreased from about 0.6 to 0.4 increasing the Reynolds number between 4×10^5^ and 8×10^5^ [14]. At Reynolds number between 2×10^6^ and 8×10^6^ the drag coefficient decreased from 0.5 to 0.1 [37]. This effect is known as “drag crisis” and it is associated with the separation of the boundary layer from the surface of the sphere [37]. Similar phenomenon can explain why the ACd of the wheelchair-sprinter system decreased with increasing speed.

Total drag ranged between 7% and 31%. However, between the catch and release phases the differences were only 3% to 7%. It is to note that the arms’ positions have a meaningful impact on the surface area. The different arms’ positions may increase the surface area and, therefore, the total drag. These results seem to be in accordance with literature. Reports in cycling and wheelchair racing noted that small variations in the rider’s positions may influence drag in about 10% [10–12]. In our case, the 12h arms position had a higher surface area in comparison with 18h and the recovery phase.

In short distances, coaches should advise their athletes to perform the propulsive phase of the stroke cycle as fast and powerful as possible. This strategy aims to reduce the winning time. In elite wheelchair racers, the recovery phase represents 65% to 67% of the stroke cycle [5, 38–41]. The remaining 33% to 35% represents the propulsive phase [5, 38–41]. During the recovery phase, athletes should maintain a good body alignment and limbs’ symmetry as much as possible to prevent an increase in the drag. The arms must be kept backwards and fully stretched. In the propulsive phase, the arms must perform the propulsion in a symmetric position and athletes must avoid spending too much time in the 12h arms position. The athletes must start a new stroke cycle as faster as possible to avoid increasing the drag. Moreover, insights on the aerodynamics can also help coaches to prescribe training sessions. E.g., designing training session or drills/sets with the goal of reducing the intra-cycle speed decay (over the recovery phase) caused by a poor body alignment.

It can be addressed as main limitations of this study: (i) the simulations were performed in static positions; (ii) one single athlete was recruited; (iii) the maximal speed selected in this study was 6.5 m/s and the average pace of the world record at the moment of the data collection was 6.62 m/s; (iv) the numerical simulations were performed assuming a temperature of 15°C and no other temperatures were tested.

## Conclusion

The obtained results shown that aerodynamics varies along the three key-moments of the stroke cycle in wheelchair racing. The position with less drag acting on the athlete-chair system was the recovery phase. The positions submitting higher drag were the catch followed-up by the release phase. These findings suggest the importance of keeping an adequate body alignment to avoid an increase in the drag force and likewise an increase of the intra-cyclic variations of the speed within the stroke cycle.

## Supporting information

S1 Dataset Partial dataset used in this research. (XLSX)

## References

[1] Forte P, Marinho DA, Morouço P, Pascoal-Faria P & Barbosa TM. Comparison by computer fluid dynamics of the drag force acting upon two helmets for wheelchair racers. AIP Conference Proceedings, AIP Publishing. 2017; 1863 (1): 520005–520008.

[2] Forte P, Barbosa TM, & Marinho DA. Technologic Appliance and Performance Concerns in Wheelchair Racing–Helping Paralympic Athletes to Excel New Perspectives in Fluid Dynamics, Chaoqun Liu (Ed.), InTech 2015; 101–121.

[3] Fuss FK. Influence of mass on the speed of wheelchair racing. Sports Engineering. 2009; 12 (1): 41–53.

[4] Cooper RA. Wheelchair racing sports science: a review. Journal of Rehabilitation research and development. 1990; 27(3): 295–312. 2205719 10.1682/jrrd.1990.07.0297

[5] Candau R, Grappe F, Menard M, Barbier B, Millet GP, Hoffman MD, et al Simplified deceleration method for assessment of resistive forces in cycling. Medicine and Science in Sports and Exercise. 1999; 31: 1441–1447. 10527317 10.1097/00005768-199910000-00013

[6] di Prampero PE. The energy cost of human locomotion on land and in water. International Journal of Sports and Medicine. 1986; 7: 55–72. 10.1055/s-2008-1025736 3519480

[7] Martin JC, Gardner AS, Barras M & Martin DT. Modelling sprint cycling using field-derived parameters and forward integration. Medicine and Science in Sports and Exercise. 2006; 38: 592–597. doi: 10.1249/01.mss.0000193560.34022.04 16540850 10.1249/01.mss.0000193560.34022.04

[8] Millet GP & Candau R. Mechanical factors of the energy cost in three human locomotions. Science & Sports. 2002; 17: 166–176

[9] Barbosa TM, Forte P, Morais JE & Coelho E. Partial contribution of rolling friction and drag force to total resistance of an elite wheelchair athlete Proc. 1st Int. Conf. in Sports Sciences & Technology (Singapore: Institute of Sports Research) 2014; 749–53.

[10] Ryschon TW, Stray-Gundersen James. The effect of body position on the energy cost of cycling. Medicine and Science in Sports and Exercise. 1991; 23(8): 949–953. 1956270

[11] McLean BD, Danaher R, Thompson L, Forges A & Coco G. Aerodynamic characteristics of cycle wheels and racing cyclists. Journal of Biomechanics. 1994; 27: 675.

[12] Paton C. Aerodynamic drag area of cyclists determined with field-based measures. Sportscience. 2006; 10: 69–71.

[13] Barbosa TM, Forte P, Estrela JE & Coelho E. Analysis of the aerodynamics by experimental testing of an elite wheelchair sprinter. Procedia Engineering. 2016; 147: 2–6.

[14] Schlichting H. Boundary Layer Theory. McGraw-Hill New York 1979.

[15] Debraux P, Grappe F, Manolova AV & Bertucci W. Aerodynamic drag in cycling: methods of assessment. Sports Biomechanics. 2011; 10(3): 197–218. doi: 10.1080/14763141.2011.592209 21936289 10.1080/14763141.2011.592209

[16] Kennedy MD & Lamo WD. Applied ergonomics of cycling performance Routledge handbook of ergonomics in sport and exercise. Hong Y. (Ed.), Routledge London 2013; 115–127.

[17] Kyle CR. Selecting cycling equipment High-Tech Cycling, Burke ER(Ed), Human Kinetics 2013; 1–48, Champaign, IL.

[18] Harun C. Aerodynamic Design of Sports Garments. Applied Aerodynamics. Jorge Colman Lerner (Ed.), InTech Rijeka 2012; 21–40.

[19] Defraeye T, Blocken B, Koninckx E, Hespel P & Carmeliet J. Computational fluid dynamics analysis of cyclist aerodynamics: Performance of different turbulence-modelling and boundary-layer modelling approaches. Journal of biomechanics. 2012; 43(12): 2281–2287. 10.1016/j.jbiomech.2010.04.038 20488446

[20] Kulfan B. Assessment of CFD predictions of viscous drag. Fluids 2000 Conference and Exhibit, Fluid Dynamics. American Institute of Aeronautics and Astronautics-2000-2391. 2000; 2–36

[21] Edwards AG, & Byrnes WC. Aerodynamic characteristics as determinants of the drafting effect in cycling. Medicine and science in sports and exercise. 2007; 39(1): 170–176. doi: 10.1249/01.mss.0000239400.85955.12 17218899 10.1249/01.mss.0000239400.85955.12

[22] Crouch TN, Burton D, LaBry ZA & Blair KB. Riding against the wind: a review of competition cycling aerodynamics. Sports Engineering. 2017; 20(2): 81–110.

[23] Bouillod A, Oggiano L, Soto-Romero G, Brunet E & Grappe F. Preliminary study: A new method to assess the effective frontal area of cyclists. 4th International Congress on Sport Sciences Research and Technology Support (IcSPORTS), Jan Cabri, João Paulo Vilas-Boas, Pedro Pezarat Correia (Eds.). Porto. 2016; 1–6.

[24] Hoffman MD, Millet GY, Hoch AZ & Candau RB. Assessment of wheelchair drag resistance using a coasting deceleration technique. American Journal of Physical Medicine & Rehabilitation. 2003; 82(11): 880–889. 10.1097/01.PHM.0000091980.91666.58 14566157

[25] Pogni M & Nicola P. Comparison of the Aerodynamic Performance of Five Racing Bicycle Wheels by Means of CFD Calculations. Procedia Engineering. 2016; 147: 74–80.

[26] Bixler B, Pease D & Fairhurst F. The accuracy of computational fluid dynamics analysis of the passive drag of a male swimmer. Sports Biomechanics. 2007; 6(1): 81–98. doi: 10.1080/14763140601058581 17542180 10.1080/14763140601058581

[27] Marinho DA, Barbosa TM, Reis VM, Kjendlie PL, Alves FB, Vilas-Boas JP, et al Swimming propulsion forces are enhanced by a small finger spread. Journal of Applied Biomechanics. 2010; 26(1): 87–92. 2010 20147761 10.1123/jab.26.1.87

[28] ANSYS Inc-. Introduction to Using ANSYS FLUENT in ANSYS Workbench: Fluid Flow and Heat Transfer in a Mixing Elbow ANSYS Fluent Tutorial Guide, Release 15.0. ANSYS Inc (eds). EUA 2013; 1–74.

[29] Winkler A & Pernpeintner A. Automated aerodynamic optimization of the position and posture of a bobsleigh crew. Procedia Engineering. 2010; 2(2): 2399–405.

[30] Defraeye T, Blocken B, Koninckx E, Hespel P & Carmeliet J. Aerodynamic study of different cyclist positions: CFD analysis and full-scale wind-tunnel tests. Journal of Biomechanics 43. 2010; 7: 1262–1268. 10.1016/j.jbiomech.2010.01.025 20171640

[31] Blocken B, Defraeye T, Koninckx E, Carmeliet J & Hespel P. CFD simulations of the aerodynamic drag of two drafting cyclists. Computers & Fluids. 2013; 71: 435–445.

[32] Griffith MD, Crouch T, Thompson MC, Burton D, Sheridan J & Brown NA. Computational fluid dynamics study of the effect of leg position on cyclist aerodynamic drag. Journal of Fluids Engineering. 2014; 136(10): 101105.

[33] Grappe F, Candau R, Belli A & Rouillon JD. Aerodynamic drag in field cycling with special reference to the Obree's position. Ergonomics. 1997; 40 (12): 1299–1311.

[34] Defraeye T, Blocken B, Koninckx E, Hespel P, Verboven P, Nicolai B & Carmeliet J. Cyclist drag in team pursuit: influence of cyclist sequence, stature and arm spacing. Journal of Biomechanical Engineering. 2014; 136(1): 011005 doi: 10.1115/1.4025792 24149940 10.1115/1.4025792

[35] Faria EW, Parker DL & Faria IE. The science of cycling: Factors affecting performance–Part 2. Sports Medicine. 2005; 35: 313–337. 15831060 10.2165/00007256-200535040-00003

[36] Barbosa TM, Morais JE, Forte P, Neiva H, Garrido ND & Marinho DA. (2017). A Comparison of Experimental and Analytical Procedures to Measure Passive Drag in Human Swimming. PloS one. 2017; 12(5): e0177038 doi: 10.1371/journal.pone.0177038 28459852 10.1371/journal.pone.0177038 PMC5411041

[37] Munson BR, Young DF & Okiishi TH. Fundamentals of Fluid Mechanics. Wiley New York 1990.

[38] Ridgway M, Pope C & Wilkerson J. A kinematic analysis of 800-meter wheelchair racing techniques. Adapted Physical Activity Quarterly. 1988; 5(2): 96–107.

[39] Gehlsen GM, Davis RW & Bahamonde R. Intermittent velocity and wheelchair performance characteristics. Adapted Physical Activity Quarterly. 1990; 7(3): 219–230.

[40] Cooper RA. An exploratory study of racing wheelchair propulsion dynamics. Adapted Physical Activity Quarterly. 1990; 7(1): 74–85.

[41] Sanderson DJ & Sommer HJ. Kinematic features of wheelchair propulsion. Journal of Biomechanics. 1985; 18(6): 423–429. 4030799 10.1016/0021-9290(85)90277-5

